# Experiences of caregivers of twins with bronchopulmonary dysplasia and retinopathy of prematurity: a phenomenological study

**DOI:** 10.15649/cuidarte.5386

**Published:** 2026-07-10

**Authors:** Sonia Esperanza Guevara Suta, Nathalya Casallas Hernández, Adriana Marcela Monroy Garzón, Olivia Margarita Narváez-Rumié, Gilma Jeannette Caraballo-Martinez, Ruth Liliana López-Cruz

**Affiliations:** 1 Respiratory Therapy Program, Health and Sports Research Group, Fundación Universitaria del Área Andina. Bogotá, Colombia. E-mail: sguevara@areandina.edu.co Fundación Universitaria del Área Andina Bogotá Colombia sguevara@areandina.edu.co; 2 Faculty of Nursing, Health Care in Individual, Family, and Social Contexts Research Group, Fundación Universitaria Sanitas, Bogotá, Colombia. E-mail: ncasallas@unisanitas.edu.co Fundación Universitaria Sanitas Bogotá Colombia ncasallas@unisanitas.edu.co; 3 Faculty of Nursing, Universidad Nacional de Colombia, Bogotá, Colombia. E-mail: amonroyg@unal.edu.co Universidad Nacional de Colombia Bogotá Colombia amonroyg@unal.edu.co; 4 Faculty of Optometry, Universidad Santo Tomás, Bucaramanga, Colombia. E-mail: olivia.narvaez@ustabuca.edu.co Universidad Santo Tomás Bucaramanga Colombia olivia.narvaez@ustabuca.edu.co; 5 Optometry Program, Fundación Universitaria del Área Andina, Bogotá, Colombia. E-mail: gcaraballo@areandina.edu.co Fundación Universitaria del Área Andina Bogotá Colombia gcaraballo@areandina.edu.co; 6 Subred Integrada de Servicios de Salud Sur, Bogotá, Colombia. E-mail: ruthlilianalopezcruz@yahoo.es Subred Integrada de Servicios de Salud Sur Bogotá Colombia ruthlilianalopezcruz@yahoo.es

**Keywords:** Twins, Premature Birth, Retinopathy of Prematurity, Bronchopulmonary Dysplasia, Caregivers, Gemelos, Nacimiento Prematuro, Retinopatía de la Prematuridad, Displasia Broncopulmonar, Cuidadores, Gêmeos, Nascimento Prematuro, Retinopatia da Prematuridade, Displasia Broncopulmonar, Cuidadores

## Abstract

**Introduction:**

The premature birth of twins with bronchopulmonary dysplasia and/or retinopathy of prematurity represents an emotional, social, and financial challenge for families.

**Objective:**

To understand the lived experiences of caregivers of premature twins diagnosed with bronchopulmonary dysplasia and retinopathy of prematurity in a Kangaroo Mother Care program in Bogotá, Colombia.

**Materials and Methods:**

A phenomenological study was conducted with seven caregivers of premature twins enrolled in a Kangaroo Mother Care program between 2020 and 2022. Virtual semi-structured interviews were transcribed and analyzed using content analysis, identifying thematic categories such as emotional experiences, access to health services, and family reorganization. Ethical considerations and confidentiality were ensured.

**Results:**

Three main categories emerged: emotional experience, caregiving challenges, and socioeconomic impact. The latter included reconfiguring family roles, financial burden, and coping strategies such as community support.

**Discussion:**

The experience of caregivers of premature twins with chronic conditions reveals a multidimensional burden that calls for comprehensive, family-centered care models with ongoing interdisciplinary support and public policies that strengthen the resilience and sustainability of caregiving.

**Conclusion:**

The importance of an interdisciplinary, family-centered, and humanized approach to neonatal care is emphasized. Future research should expand the sample and explore targeted interventions for this population.

## Resumen

**Introducción:**

El nacimiento prematuro de gemelos con displasia broncopulmonar y/o retinopatía del prematuro representa un desafío emocional, social y económico para las familias.

**Objetivo:**

Comprender las experiencias vividas de cuidadores de gemelos prematuros diagnosticados con displasia broncopulmonar y retinopatía en el contexto de un Programa Madre Canguro en Bogotá, Colombia.

**Materiales y métodos:**

Se realizó un estudio fenomenológico con siete cuidadores de gemelos prematuros participantes en el Programa Madre Canguro entre 2020 y 2022. Las entrevistas semiestructuradas virtuales se transcribieron y analizaron mediante análisis de contenido, identificando categorías temáticas como experiencia emocional, acceso a servicios de salud y reorganización familiar. Se aseguraron aspectos éticos y confidencialidad.

**Resultados:**

Emergieron tres categorías principales: Experiencia emocional, desafíos en el cuidado e impacto socioeconómico: Reconfiguración de roles familiares, carga económica y estrategias de afrontamiento como el apoyo comunitario.

**Discusión:**

La experiencia de cuidadores de gemelos prematuros con patologías crónicas revela una carga multidimensional que demanda modelos de atención integrales, centrados en la familia, con apoyo interdisciplinario continuo y políticas públicas que fortalezcan la resiliencia y sostenibilidad del cuidado.

**Conclusiones:**

Se destaca la importancia de un enfoque interdisciplinario y humanizado en la atención neonatal, centrado en las familias. Futuras investigaciones deberían ampliar la muestra y explorar intervenciones específicas para esta población.

## Resumo

**Introdução:**

O nascimento prematuro de gêmeos com displasia broncopulmonar e/ou retinopatia da prematuridade representa um desafio emocional, social e econômico para as famílias.

**Objetivo:**

Compreender as experiências vividas por cuidadores de gêmeos prematuros diagnosticados com displasia broncopulmonar e retinopatia no contexto de um Programa de Cuidado Materno Canguru em Bogotá, Colômbia.

**Materiais e métodos:**

Foi realizado um estudo fenomenológico com sete cuidadores de gêmeos prematuros participantes do Programa de Cuidado Materno Canguru entre 2020 e 2022. Entrevistas virtuais semiestruturadas foram transcritas e analisadas por meio de análise de conteúdo, identificando categorias temáticas como experiência emocional, acesso a serviços de saúde e reorganização familiar. As considerações éticas e a confidencialidade foram asseguradas.

**Resultados:**

Três categorias principais emergiram: experiência emocional, desafios do cuidado e impacto socioeconômico: reconfiguração dos papéis familiares, ônus econômico e estratégias de enfrentamento, como o apoio da comunidade.

**Discussão:**

A experiência dos cuidadores de gêmeos prematuros com doenças crônicas revela um fardo multidimensional que exige modelos de cuidado abrangentes e centrados na família, com apoio interdisciplinar contínuo e políticas públicas que fortaleçam a resiliência e a sustentabilidade do cuidado.

**Conclusões:**

Destaca-se a importância de uma abordagem interdisciplinar e humanizada para o cuidado neonatal, centrada nas famílias. Pesquisas futuras devem ampliar o tamanho da amostra e explorar intervenções específicas para essa população.

## Introduction

Premature birth is a complex event that poses multiple challenges for the newborn and the family. Each year, approximately 15 million infants are born prematurely, making it one of the leading causes of neonatal morbidity and mortality worldwide^1^. Among the most common complications are bronchopulmonary dysplasia (BPD) and retinopathy of prematurity (ROP), which may result in long-term respiratory and visual impairments and require specialized medical follow-up^2^,^3^.

Caring for a child with BPD and/or ROP entails a significant emotional, social, and financial impact on the family, due to prolonged hospitalizations, complex treatments, and uncertainty regarding their children’s development^4^. Parents of preterm infants experience high levels of stress, anxiety, and depression, arising from their children’s vulnerability and the need to assume an active caregiving role^5^-^7^. Studies report that approximately 40% of parents experience depressive symptoms and around 50% experience anxiety symptoms in the immediate period following premature birth, while up to three-quarters report high levels of overall stress^8^,^9^. This burden is intensified in the case of premature twins, as caregiving demands are doubled and challenges in family organization and access to support resources increase^10^.

Early intervention programs, such as Kangaroo Mother Care (KMC), have shown benefits for the prognosis of preterm infants and for strengthening parenting^11^. However, evidence on their impact on families of twins with BPD and/or ROP is limited. Previous literature has primarily focused on the mother-infant relationship and psychosocial risk factors, overlooking the experience of simultaneously caring for two children with complex chronic conditions, the challenges of family reorganization, and the levels of stress this entails^12^. Furthermore, it is essential to understand how family members experience the transition from hospital to home and which coping strategies they develop to manage the emotional and logistical burden of care.

From a phenomenological perspective, exploring the experiences of these caregivers provides insights into the meanings they ascribe and their needs for emotional, social, and health care support in this context. Accordingly, this study aimed to understand the experiences of caregivers of premature twins with bronchopulmonary dysplasia and retinopathy of prematurity in a Kangaroo Mother Care program in Bogotá, Colombia.

## Materials and Methods

**Study design**

A qualitative study with a phenomenological approach was conducted to understand the experiences of caregivers of twins with BPD and/or ROP enrolled in a KMC program in Bogotá between 2020 and 2022. This approach enabled the exploration of the meanings underlying their lived experiences^13^. As this was a qualitative study, the Consolidated Criteria for Reporting Qualitative Research (COREQ) guidelines were followed^14^.

The sample consisted of seven caregivers of premature twins, selected through convenience sampling supported by the program guidelines, based on their experience and eligibility. Inclusion criteria were caregivers aged 18 years or older; infants born at less than 37 weeks’gestation; and active participation in the KMC program for 3 to 24 months. Finally, caregivers of children with cognitive impairments, congenital malformations, special needs, or health conditions that could limit participation were excluded, as well as those who declined to participate in the study.

**Data collection**

Data were collected between 2020 and 2022, a period marked by the pandemic, which led to significant adjustments in the KMC program. Caregivers were initially contacted by telephone, and the study was briefly introduced, and potential risks were explained. All family members agreed to participate voluntarily. Subsequently, semi-structured interviews were conducted, with questions adapted to caregivers’ level of understanding to explore their emotional life experiences, caregiving experiences, and the difficulties encountered during the process. The average duration of each interview was 45 minutes.

A total of seven virtual interviews were conducted by the principal investigator, who has extensive experience in the comprehensive care of preterm infants and their families. No pilot testing was conducted, nor was it necessary to repeat interviews, as the accounts obtained were sufficient for the analysis and understanding of the phenomenon under study.

**Data analysis**

**Figure 1:**
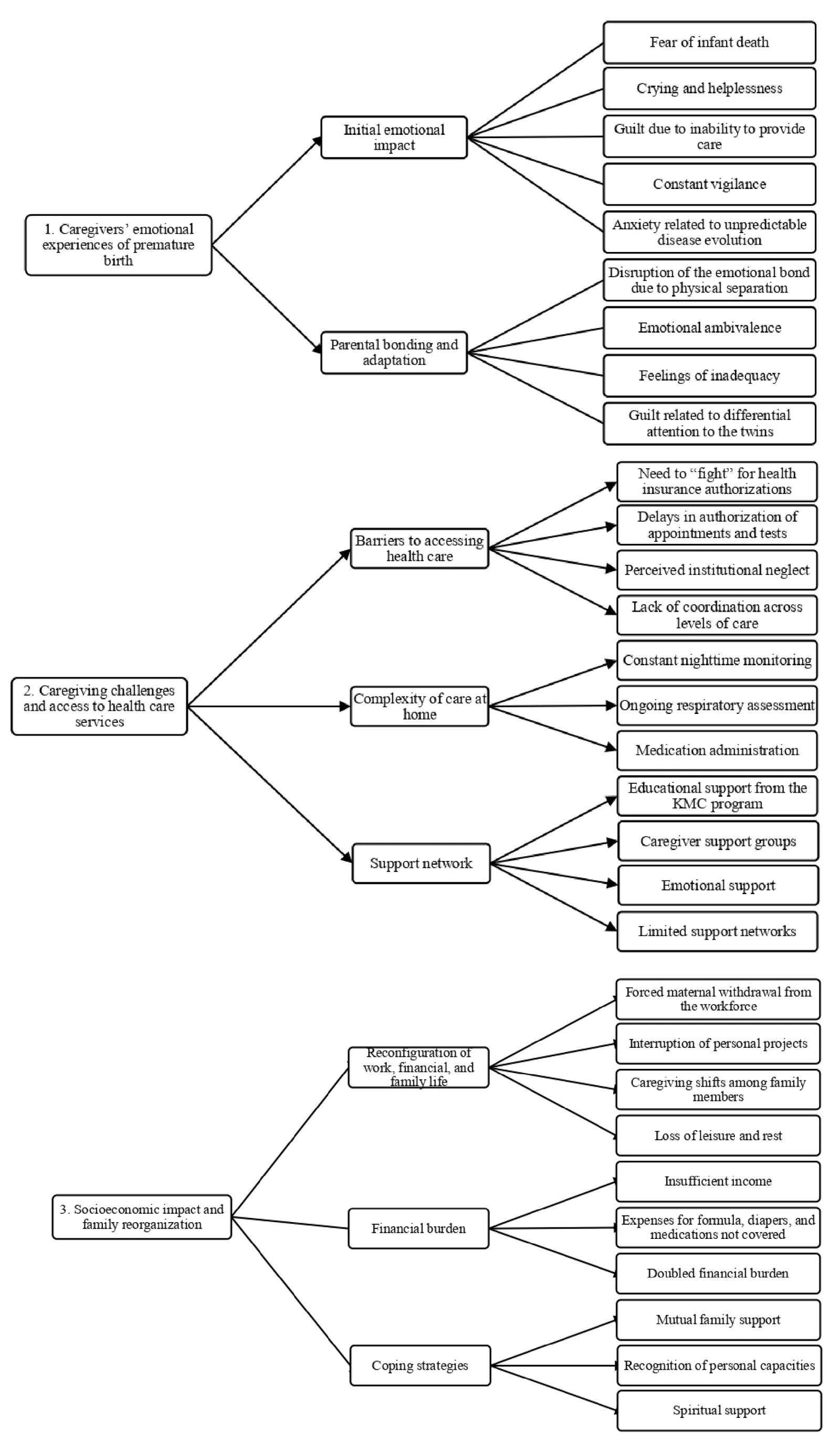
Hierarchical coding tree

The interviews were audio-recorded, transcribed verbatim, and shared with participants, who returned them without requesting any modifications. Data were managed using ATLAS.ti software. Data analysis followed Bardin’s content analysis approach through an inductive process that included the phases of pre-analysis, coding, and categorization^15^,^16^. Coding was performed independently by two co-investigators, who conducted iterative readings of the transcripts to identify relevant meaning units. Subsequently, a hierarchical coding tree was developed from codes formulated as interpretive expressions, which condensed the shared meanings identified in the accounts and facilitated the organization of the material into subcategories and categories. These categories enabled the identification of recurring patterns and, in turn, their grouping into core categories that comprehensively explained the overall experience, as presented in the Results section. Themes were derived inductively, emerging directly from the discourse until theoretical saturation was reached in the seventh interview, when redundancy in the accounts and a deep understanding of the phenomenon under study became evident, as shown in Figure 1.

To ensure confidentiality, participants were identified using codes (P1, P2, P3, and so on). For triangulating qualitative data, the literature review and semi-structured interviews were used as complementary data sources. Methodological triangulation allowed comparison of emerging findings from caregivers’ accounts with existing theoretical knowledge on the experience of caregiving in preterm multiple births, thereby strengthening the credibility and trustworthiness of the results. Although observation diaries were initially considered as a third source of triangulation, pandemic-related constraints precluded their inclusion; therefore, greater emphasis was placed on comparative analysis between the primary data and the specialized literature. To ensure confidentiality, the accounts were coded and organized into an anonymized qualitative dataset, available in Mendeley Data^17^.

**Ethical considerations**

In accordance with Resolution 8430 of 1993 issued by the Colombian Ministry of Health, this study was classified as minimal risk, as participants could experience discomfort or emotional distress^18^. Ethical approval was obtained from the Research Ethics Committee of the Subred Sur (approval No. 238-10032023). Following participants’ verbal agreement, informed consent was obtained, ensuring opportunities to address any questions.

## Results

 Seven caregivers of premature twins (parents and grandmother) were interviewed, with ages ranging from 20 to 53 years. Of these, 85.71% (n=6) were in socioeconomic stratum 1 (the lowest level in Colombia’s socioeconomic classification system), 71.42% (n=5) lived in rented housing, and 100% (n=7) reported having support networks. The number of prenatal care visits ranged from 2 to 10 Table 1.

**Table 1:** Individual characteristics of study participants

Code	Kinship	Age (years)	Education	Occupation	Socioeconomic stratum	Housing	Prenatal care visits	Support network
P1	Mother	29	Incomplete primary education	Homemaker	2	Rented	6	Yes
P2	Mother	24	Incomplete secondary education	Informal vendor	1	Rented	2	Yes
P3	Mother	38	Incomplete primary education	Homemaker	1	Rented	10	Yes
P4	Mother	20	Incomplete secondary education	Student	1	Rented	2	Yes
P5	Grandmother	52	Incomplete primary education	Homemaker	1	Rented	Not applicable	Yes
P6	Mother	33	Incomplete primary education	Homemaker	1	Owned	5	Yes
P7	Father	53	Incomplete primary education	Farmer	1	Owned	Not applicable	Yes

Source: Kangaroo Mother Care Program database, Subred Sur, 2020-2022.

From the analysis, three categories and their respective subcategories emerged, derived from the codes identified in participants’ accounts Table 2. 

**Table 2:** Summary of the process of category and subcategory development

Theme	Category	Subcategory	Code
Caregivers’ experience	Parents’ emotional experience of premature birth	Initial emotional impact Parental bonding and adaptation	Reactions of shock, fear, and distress upon receiving the diagnosis of extreme prematurity. Process of strengthening attachment and adapting to new responsibilities.
Care	Caregiving challenges and access to health care services	Barriers to accessing health care Complexity of care at home	Difficulties accessing appointments, transportation, and specialized services. Management of oxygen therapy, specialized feeding, and adjunct therapies.
Consequences	Socioeconomic impact and family reorganization	Reconfiguration of work, financial, and family life Coping strategies	Adaptation of routines, redistribution of roles, and workforce withdrawal. Ways in which families cope with and overcome challenges.

**Category 1. Caregivers’ emotional experience of premature birth**

The premature birth of twins with chronic conditions such as BPD and/or ROP constitutes a disruptive experience. Participants’ accounts reflect a transition from the expectation of a normal pregnancy to a complex and highly demanding medical reality. The emotions described do not follow a linear course but rather are intertwined and evolve.

**Initial emotional impact:** Parents described twin delivery and the clinical diagnosis as a disruptive experience, characterized by a sense of loss of control, vulnerability, fear, and distress in the face of the possibility of losing their children. This perceived imminent threat marked the beginning of a complex emotional process.

*“When they told me my babies had to stay in the ICU, I felt like my world was falling apart. I didn’t know what to do—I just cried.” (P2)”*

Infants’ body language, such as minimal movements, extreme fragility, and technological dependence, generated an affective shock that some caregivers described as paralyzing.

*“It was really hard to see them so tiny and hooked up to all those wires. I felt helpless, like there was nothing I could do for them.” (P1)*

Birth under these conditions initially limits parents in their role as active caregivers, leading to feelings of guilt, inadequacy, or uselessness that affect bonding. In multiple pregnancies, this impact is amplified as parents face the challenge of attending to the pain, fragility, and needs of both infants.

*“When they told me it was a high-risk pregnancy, I was really scared because they said I had to be extremely careful and stop working.” (P6)*

In turn, the unpredictable clinical course of the twins led to a state of constant vigilance, with limited physical and emotional rest and a persistent climate of anxiety. In this context, participants drew on various emotional, spiritual, and social strategies to cope.

*“At first they told me they weren’t going to make it because they were so tiny, but I held on to hope, and now they’re here with me.” (P7)*

In summary, the initial emotional impact is expressed as acute distress, marked by the perceived life-threatening risk to their children or grandchildren, the perceived inability to fulfill the caregiving role, the disruption of expectations, and entry into a highly technological hospital environment that depersonalizes the experience of birth, making it necessary to re-signify the meaning of being a caregiver under extreme conditions.

**Parental bonding:** In preterm multiple births, parental bonding was a progressive process marked by emotional ambivalence. Unlike what occurs under typical conditions, parents of preterm twins with BPD and/or ROP experienced a disruption or delay in emotional bonding due to physical separation, the medicalization of care, and the constant fear of loss.

*“At first I was afraid to hold them—they were so tiny and fragile. But little by little I learned to trust myself and to believe they’re strong too. I felt like I wasn’t capable, but over time I realized they need to feel my love and my warmth.” (P5)*

The KMC program played a fundamental role in this process. Guidance and support sessions enabled parents to take an active role in their infants’ care, transforming fear into competence and emotional distance into closeness.

*“When they showed us how to hold them and what to do, I felt like I was a mom again. I wasn’t just a bystander in the hospital anymore.” (P2)*

In the specific case of twins, this process was doubled and took on distinct nuances. Some parents expressed concern about forming unequal bonds with each child, particularly when one twin had a more complex clinical course than the other.

*“Sometimes I paid more attention to the one who was more fragile, and then I’d feel guilty for not being the same with the other. You don’t want there to be any differences, but it’s hard.” (P7)*

In sum, parental bonding in this context was neither immediate nor spontaneous but rather the result of a process of emotional reconstruction, kinesthetic learning, and redefinition of the maternal or paternal role. Premature birth did not prevent bonding; rather, it reshaped it into more conscious, engaged, and transformative forms.

**Category 2. Caregiving challenges and access to health care services**

Caring for premature twins with chronic conditions is embedded in a daily reality shaped by practical challenges. This category captures the tensions caregivers experience in accessing healthcare, the complexities of specialized home-based care, and how they perceive the presence or absence of support from institutions and formal and informal networks.

**Barriers to accessing health care: **Participants’ accounts indicate that one of the greatest difficulties was timely, continuous, and effective access to healthcare services. The barriers identified were related not only to structural shortcomings of the system but also to a subjective experience of abandonment, invisibility, and emotional strain.

*“We’ve had to fight with the insurance company to get authorizations. If you don’t push, they don’t do anything.” (P2)*

Caregivers reported that specialist appointments, diagnostic tests, and required therapies were not scheduled promptly, as needed. These delays were perceived as a direct risk to the infants’ health, generating anxiety and feelings of helplessness.

*“Sometimes they’d give me appointments way too late, and I had to keep pushing to get them seen sooner because they can’t wait that long.” (P4)*

Access barriers also included logistical challenges, such as transportation to attend appointments, particularly for infants dependent on oxygen or with medically fragile conditions. In contexts of financial vulnerability, these limitations were exacerbated by the inability to cover associated costs, compromising adherence to follow-up care.

Complexity of care at home: Hospital discharge of preterm twins marked, for caregivers, the beginning of a new phase characterized by high demands, fears, and highly specialized responsibilities. Participants’ accounts show that the home, rather than being a place of rest and safety, became an extension of the clinical setting.

*“It’s really hard. You have to keep an eye on the oxygen, make sure it doesn’t come loose, make sure they’re eating well. You never really sleep peacefully.” (P1)*

Parents assumed the role of specialized caregivers without prior professional training. Handling medical devices, such as oxygen cylinders, nasal cannulas, or inhalers, and administering medications with strict dosing and schedules became part of the daily routine, keeping a constant state of alertness.

*“I’m scared that something could happen at any moment and I wouldn’t know what to do. Every night I check that they’re breathing okay—I don’t sleep.” (P4)*

This continuous vigilance had a significant impact on caregivers’ physical and mental health, with reports of exhaustion and persistent anxiety about the possibility of an emergency occurring at home without being able to respond in time. This fear was compounded by the need to care for two infants with different needs simultaneously, doubling the demands and caregiving burden.

*“After the hospitalization, one of them needed oxygen for two months. It was really hard because he needed more attention. The other baby was doing better, but if one got sick, I’d end up neglecting the other.” (P3)*

Despite these challenges, caregivers established routines, learned through observation, relied on family support networks, and developed practical, everyday knowledge that enabled them to sustain their infants’ lives. Participants’ accounts indicate that the close, educational support provided by the KMC team was key to strengthening confidence in home-based care by providing technical knowledge, building confidence, and fostering a sense of not being alone in facing this challenge.

*“It helped us a lot that a nurse from the program explained every step. Without that support, we wouldn’t have known what to do.” (P6)*

In addition to the healthcare team, participants identified other sources of emotional and practical support that were essential for sharing responsibilities, providing emotional support, and offering resources such as food or transportation assistance.

*“In the support group, I met other moms going through the same thing. It made me feel like I wasn’t alone, like others were dealing with it too.” (P3)*

However, support was not uniform. Some parents reported feeling alone, with insufficient support from the health system or limited family networks. This situation highlighted that access to emotional and practical support is shaped by structural factors, including socioeconomic status, available support networks, and institutional responsiveness.

**Category 3. Socioeconomic impact and family reorganization**

This category encompasses the adaptations and trade-offs families experience at the financial and household levels, where caregiving requires the redistribution of roles, changes in routines, and a reduction in household income. 

**Reconfiguration of work and family life:** The premature birth of twins with chronic conditions entailed a profound transformation of work and domestic dynamics. This was not a simple adjustment but a rupture with the previous way of life, requiring difficult decisions, personal sacrifices, and a reorganization of roles centered on the care of the children.

*“I had to quit my job because there was no one to take care of them. Now we’re living on just one income, and it’s been really hard.” (P2)*

Mothers, in most cases, assumed a central caregiving role, which involved leaving their jobs, interrupting their studies or personal activities, and dedicating themselves exclusively to the twins’ care.

*“Before I knew it was a high-risk pregnancy, I worked for two months, but I had to stop for my health and the babies’. My husband has two jobs and can’t help me with the kids, so my mom is the one who helps me.” (P3)*

Family daily life was disrupted by routines centered on the twins’ medical needs. Household tasks, relationships with other children, rest, and leisure were reconfigured or disappeared. The home was transformed into a therapeutic and highly monitored space, where each family member had to adapt to a new order shaped by the infants’ fragility.

*“My wife and I had to figure out a schedule to take turns at the hospital and at home, but it ended up affecting our finances.” (P7)*

At the same time, caring for two infants resulted in a sustained financial burden, intensified by the complexity of providing simultaneous care. This burden involved not only increased financial costs but also persistent financial strain, where the fear of being unable to meet needs coexisted with an unconditional commitment to ensuring the children’s survival and well-being.

*“The money just isn’t enough. The diapers, the special formula, the medications… it’s too much, and sometimes we don’t know how we’re going to pay for it.” (P2)*

The main sources of expenditure reported by participants included specialized formula, large quantities of diapers, frequent transportation to medical appointments and hospitals, medications not covered by health insurance, adjunct therapies, follow-up tests, and minor home modifications to ensure safe conditions.

*“We spend everything on the treatments, and even though some things are covered, there’s still a lot we have to pay for ourselves.” (P1)*

In sum, the financial burden of caring for premature twins with BPD and/or ROP emerges as a factor that exacerbates family vulnerability, undermines emotional stability, and highlights the need for public policies responsive to the complexity of these situations.

**Coping strategies:** This subcategory illustrates how families strengthen their capacity to cope with the challenges of having a newborn with special health care needs. Mutual support and solidarity-based networks became spaces for emotional support and practical assistance, facilitating more effective coping with stress and uncertainty.

*“We’ve learned to be strong as a family. We support each other a lot, and also connect with other moms in the program.” (P5)*

Participants’ accounts indicate a process of personal and family transformation in which families initially feel overwhelmed and vulnerable but, over time, come to recognize their inner strength and collective capacity to overcome challenges. This resilient growth becomes a source of hope and motivation to move forward despite difficulties.

*“At first I didn’t think I could handle this, but now I see how far we’ve come and realize we’re stronger than we ever imagined.” (P6)*

This strengthening process not only improves families’ quality of life but also fosters a deep sense of hope and empowerment. Thus, coping strategies and mutual support are consolidated as essential pillars in the path toward emotional recovery and overall well-being of families in the context of specialized neonatal care.

## Discussion

The findings of this study provide an in-depth understanding of the lived experiences of caregivers of premature twins with BPD and/or ROP. These chronic and complex conditions impose a significant burden not only on the health of the newborns but also on the emotional, social, and financial domains of family life^4^.

The process of parental bonding emerges as a dynamic and ambivalent phenomenon in which hope is intertwined with fear and guilt, shaping a complex emotional landscape that affects family adaptation^5^,^9^. The convergence of preterm multiple births with severe conditions amplifies uncertainty and stress, requiring parents to manage the intensive care of two children in highly medicalized hospital settings^19^. This situation increases psychological and emotional vulnerability, as previously documented, and is further expanded here by highlighting the specific features of parental bonding in twins and the affective demands it entails^20^,^21^.

The KMC program emerges as a key strategy to facilitate early contact and active parental involvement, promoting bonding and caregiving competence^11^. However, caring for twins requires specific adaptations to ensure balanced care and to avoid disparities in bonding or caregiving. This aspect warrants greater attention in clinical practice and future research.

Uncertainty, perceived as a constant source of stress, is addressed through strategies that include spiritual and family support, as well as communication with health care professionals^22^. This multifaceted coping strengthens resilience and adaptive capacity, facilitating the management of prolonged neonatal care. However, limited access to psychosocial services and fragmented care create significant gaps, exacerbating isolation and vulnerability. This finding is consistent with previous reports that underscore the need for interdisciplinary, continuous programs integrating emotional, social, and clinical support^23^.

At a structural level, participants’ accounts indicate barriers to accessing quality health care, including delays in scheduling appointments, lack of coordination across levels of care, and lack of individualized follow-up^24^. These obstacles reflect systemic issues that hinder continuity of care, which is essential in BPD and ROP due to the need for regular monitoring and specialized treatments. The perceived lack of support and the ongoing struggle to access basic healthcare services highlight the need to strengthen health systems through family-centered models that ensure humanized, coordinated, and comprehensive care, addressing both clinical and psychosocial needs^21^.

Likewise, preterm multiple births profoundly affect family structure and dynamics, requiring a reorganization of roles and responsibilities that, in many cases, entails significant financial and social sacrifices. Job loss, increased direct and indirect costs, and adaptations within the home reflect the absence of adequate social support and of public policies that recognize the magnitude of these burdens. These findings are consistent with international studies indicating that premature birth can destabilize family well-being and lead to long-term consequences for caregivers’ quality of life. In this regard, health programs should incorporate mechanisms for financial support, alternative caregiving services, and psychological support to alleviate family burden and promote the sustainability of the caregiving environment^25^,^26^.

Finally, the findings of this study provide valuable insights for clinical practice and the development of public policies aimed at improving the quality of life of families of preterm infants with complex conditions. The need for an interdisciplinary approach is emphasized, integrating clinical management, emotional and social support, and adapting programs such as KMC to address the specific needs of preterm multiple birth^27^. In addition, the importance of future research is highlighted to explore family experiences across diverse cultural and socioeconomic contexts and to evaluate interventions designed to strengthen resilience and long-term well-being^28^.

This study has limitations that should be considered when interpreting the findings. The small sample size and recruitment from a single KMC program in Bogotá limit the transferability of the results to other cultural, institutional, or socioeconomic contexts. In addition, virtual data collection during the pandemic restricted the observation of nonverbal cues important for phenomenological interpretation. Finally, as a cross-sectional study focused on a specific point in the caregiving process, it did not allow exploration of changes in parental bonding or the sustainability of coping strategies over time^5^,^6^.

## Conclusions

The experience of family members of twins with bronchopulmonary dysplasia and retinopathy of prematurity reveals a complex clinical, emotional, and social burden. Although the Kangaroo Mother Care program strengthens parental bonding and caregiving, it requires adaptations to address the demands of simultaneous care. Barriers to access, along with the lack of psychosocial and financial support, increase family vulnerability, underscoring the need to strengthen health systems through comprehensive, family-centered approaches and to advance public policies and research that address these needs.

Finally, this study reaffirms the central role of the interdisciplinary neonatal health care team in the comprehensive, humanized care of premature twins with chronic conditions and their families. Their role extends beyond clinical and technical tasks to include emotional support, caregiver education, and the coordination of support networks. The findings highlight these professionals as key actors in the consolidation of family-centered care models, capable of responding to the complexity of preterm multiple births and contributing significantly to improving the quality of life of both the newborns and their families.
